# Enhancing *Candida auris* Surveillance in High-Risk Settings by Implementing a High-Throughput Molecular Assay on the Hologic Fusion Open Access Platform

**DOI:** 10.3390/jof10040285

**Published:** 2024-04-12

**Authors:** Filipe M. Cerqueira, Jennifer Bertsch, Mary Ann DeMaet, Teresa York, April McDougal, Janak A. Patel, Ping Ren

**Affiliations:** Department of Pathology, University of Texas Medical Branch, Galveston, TX 77555, USA; fmcerque@utmb.edu (F.M.C.); jabertch@utmb.edu (J.B.); mademaet@utmb.edu (M.A.D.); teyork@utmb.edu (T.Y.); apmcdoug@utmb.edu (A.M.); jpatel@utmb.edu (J.A.P.)

**Keywords:** *Candida auris*, surveillance, high-throughput, infection control

## Abstract

*Candida auris*, a resilient pathogenic yeast with frequent multidrug resistance, presents a persistent challenge in healthcare settings. The timely identification of *C. auris* is crucial for infection control and prevention, especially in facilities facing unique hurdles, such as our institution, which serves four major hospitals and approximately 80% of the Texas inmate population. Understaffing, communal living, and financial constraints exacerbate infection control issues. To address common staff shortages, streamline testing services, and enhance testing efficiency, there was a pressing need for rapid and high-throughput detection of *C. auris*. This study presents the validation and utility of an assay implemented on the Hologic Fusion Open Access platform using samples collected from high-risk patients’ axilla and groin areas, as well as environmental swab samples from patient rooms. Our assay complemented efforts to control *C. auris* outbreaks within our healthcare system, providing valuable insights into its presence within surveillance samples. This assay demonstrated the value of high-throughput molecular detection platforms in challenging healthcare environments by aiding infection preventionists in containing the spread of *C. auris* and preventing nosocomial infections. Our research contributes essential data on the suitability and performance of the Hologic Fusion Open Access platform for *C. auris* detection. These findings hold significant implications for enhancing surveillance and control measures in high-risk settings, making a significant impact on the field of infection control and prevention.

## 1. Introduction

Since its initial discovery in Japan in 2009, *Candida auris* has emerged as a formidable fungal pathogen as it often displays multidrug resistance and is associated with widespread global outbreaks [1,2,3,4,5]. Its capacity to cause invasive nosocomial infections with alarmingly high mortality rates within healthcare facilities is of particular concern [6,7]. Unfortunately, the most vulnerable individuals to systemic *C. auris* infection encompass patients recently admitted into the intensive care units (ICUs), patients with chronic conditions like diabetes and renal failure, and those with a recent history of antibiotic exposure or surgery [8,9]. Additionally, *C. auris* has demonstrated the ability to persist on environmental surfaces even after cleaning with antiseptics and can be transmitted from asymptomatic colonized patients to immunocompromised individuals who are particularly vulnerable to infection [10,11,12,13]. Moreover, *C. auris* possesses the ability to form adherent biofilm communities on various clinically important substrates, including catheter material [9]. Following guidelines set forth by the Centers for Disease Control and Prevention (CDC), our institution introduced a comprehensive *C. auris* screening protocol for high-risk patients in November 2021, employing the routine fungal culture method (https://www.cdc.gov/fungal/candida-auris/identification.html (accessed on 21 November 2021)). As per the CDC’s recommendations, high-risk patients are defined as those who have received care within the preceding 90 days at a long-term acute care facility (LTAC), nursing home, rehabilitation center, or skilled nursing facility (SNF). These high-risk patients are included in our surveillance program, with an automated admission order in the electronic medical record for *C. auris* screening by swabbing the axilla and groin areas since these sites are the most common and consistent sites of colonization, although other body sites may also be colonized [14,15]. These patients were placed in isolation until the availability of *C. auris* culture results.

However, in July 2022, a case of *C. auris* fungemia was diagnosed in our adjoining prison hospital. In our pre-admission screening protocol, the prisoner population was not included as it was not considered to be a high-risk group. Subsequently, we conducted mass screening of 344 patients within the prison hospital, leading to the identification of additional patients colonized with *C. auris.* During the investigation and management of this outbreak, the infection control team required terminal cleaning of the rooms with bleach, followed by environmental cultures for *C. auris* to document the effectiveness of the cleaning process in rooms previously occupied by *C. auris*-positive patients. The rooms were released for patient occupancy only if the cultures were negative. The collection-to-results turnaround time (TAT) was three to five days. This delay was significant as it adversely affected patient care during the outbreak.

To address this issue, we developed and validated an automated real-time PCR (RT-PCR) laboratory-developed test (LDT), hereby referred to as the CAURIS-PCR assay. CAURIS-PCR was designed to detect *C. auris* DNA in high-risk patients, specifically collecting specimens from the axilla and groin, as well as from environmental swabs taken from eight high-touch areas after discharging *C. auris*-colonized patients from their rooms.

The CAURIS-PCR assay was developed and performed on the Hologic^®^ Open Access Fusion^®^ (Marlborough, MA, USA) platform, with the primary goal of expediting the detection process and ultimately improving patient care during outbreaks of *C. auris* infections.

## 2. Methods

Sample collection: Sample collection procedures were standardized for consistency and efficacy [15]. ESwab™ (COPAN Diagnostics, Murrieta, CA, USA) was used for the specimen collection.

For axilla/groin samples, a sterile swab was utilized to swipe back and forth 5 times per armpit. Then, using the same swab, the left and right groin skin surfaces were each swiped 5 times. The swab was then carefully placed in Amies media for subsequent culture or CAURIS-PCR assay (https://www.cdc.gov/fungal/candida-auris/c-auris-patient-swab.html (accessed on 21 November 2021)).

Immediately following the discharge of a *C. auris*-positive patient, our established protocol for room cleaning and environmental sample collection was initiated. Environmental sample collection was conducted post-room disinfection using 10% bleach. If *C. auris* was detected following the initial bleach disinfection, UV light exposure was performed alongside a second round of bleach cleaning before sample collection. Post-disinfection, a sterile swab moistened with sterile water was employed to sample high-touch areas within the hospital room. These areas typically included the door, bed, patient chair, IV pole, tables, sink area, bathroom, and nurse workstation. Each swab was individually placed in Amies media for subsequent culture or CAURIS-PCR assay. This meticulous process ensured comprehensive surveillance of potential *C. auris* contamination within the healthcare environment.

*Candida auris* culture: Approximately 250 µL of the Amies media in ESwab™ was plated on HardyCHROM™ Candida + auris chromogenic media (Hardy Diagnostics, Santa Maria, CA, USA). Plates were incubated at 37 °C for 72 h and screened for teal to teal-green colonies with “bullseye” centers that were positive for UV fluorescence. All potential *C. auris* colonies were further confirmed by Bruker matrix-assisted laser desorption ionization-time of flight mass spectrometry (MALDI-TOF MS).

Nucleic acid capture and elution: Prior to processing and testing on the Panther Fusion^®^ system (Marlborough, MA, USA), 300 µL of Amies media from either the axilla/groin or environmental ESwab specimens were transferred into Aptima^®^ Tubes which were pre-aliquoted with equal volume (300 µL) of specimen transport media (STM). STM lyses cells, releases target nucleic acids, and protects the nucleic acids from degradation during storage. The Internal Control-X (IC-X) was added to each specimen, including controls via the addition to the Panther Fusion^®^ Capture Reagent-X (FCR-X) (Marlborough, MA, USA). The IC-X in the reagent monitors the various stages of specimen processing, amplification, and detection. Capture oligonucleotides engage in hybridization with organism-specific nucleic acid present in the specimens. Subsequently, hybridized nucleic acids are separated from the specimen via the application of a magnetic field. Successive wash steps effectively remove extraneous components from the reaction tube. In the final elution step, purified nucleic acids are carefully released. This comprehensive process ensures the isolation of total nucleic acids from the specimens.

Elution transfer and real-time PCR: In the elution transfer step, the purified nucleic acid is carefully transferred to a Panther Fusion^®^ reaction tube preloaded with oil and a reconstituted master mix. Target amplification occurs via multiplex real-time PCR, which is driven by specific forward and reverse primers and probes, all designed to simultaneously amplify, detect, and discriminate multiple target types.

Development of LDT protocol on Panther Fusion: The Hologic Open Access Software (Version 2.1.2.1) was used to develop an LDT protocol on the Panther Fusion system. For this purpose, we employed the “X” Fusion Capture and Enhancer reagent combination. In addition, we utilized Hologic internal control primers and probes, which were designed for detection in the Quasar 705 channel, ensuring the reliability of the testing process. To specifically identify *C. auris*, we incorporated primers and probes designated by the CDC in the FAM channel, allowing us to precisely and selectively detect *C. auris*. The *C. auris* primers and probes are as follows: 5′-CAG ACG TGA ATC ATC GAA TCT-3′, 5′-TTT CGT GCA AGC TGT AAT TT-3′, 5′-/56-carboxyfluorescein (FAM)/AAT CTT CGC/ZEN/GGT GGC GTT GCA TTC A/3IABkFQ/-3′ [16]. The thermocycler protocol is as follows: 1 cycle of 95 °C for 2 min, 45 cycles of 95 °C for 5 s, and 60 °C for 30 s. The criteria for identifying positive samples were defined as exceeding 2000 fluorescence value (FLU) before the Cycle Threshold (Ct) of 40. In our screening process, both culture-negative and culture-positive samples were examined. Our analysis revealed that a Ct value of 40 represents the optimal point on the amplification curve of CAURIS-PCR. Importantly, this specific Ct value demonstrated the closest correlation with clear positive and negative results in our assessment.

Validation of the CAURIS-PCR assay: For the contrived samples, yeast isolates were first subcultured on Sabouraud Dextrose Agar (SAB) (Remel, Lenexa, KS, USA) and subsequently resuspended in sterile saline to achieve turbidity equivalent to a 0.5 McFarland standard. Then, 1:10 serial dilutions were performed using either sterile Amies media (Rmbio, Missoula, MT, USA) or pooled *C. auris*-negative ESwab axilla/groin or environmental samples as matching matrices. A 100 µL volume of the suspensions with expected concentrations between 300 and 3000 CFU/mL was plated in triplicate on SAB. After 2 days of incubation at 37 °C, colonies were counted. Concentrations were back-calculated from dilutions that yielded an average colony count ranging from 30 to 300 per plate. To assess the assay’s reproducibility, negative (uninoculated Amies media), low *C. auris* concentration (677 CFU/mL), and high *C. auris* concentration (6.77 × 10^4^ CFU/mL) samples were tested in triplicates by two technologists on three different days. Additionally, analytical specificity was evaluated using various other yeast species, including *C. duobushaemulonii*, *C. haemulonii*, *C. kruseii*, *C. lusitaniae*, *Kodamaea ohmeri*, and *Saccharomyces cerevisiae*. Furthermore, the assay’s ability to detect currently circulating clades of *C. auris* was assessed using five strains from different clades provided by the CDC (Table 1) (https://wwwn.cdc.gov/ARIsolateBank/Panel/PanelDetail?ID=2 (accessed on 12 June 2023)).

To evaluate the accuracy of CAURIS-PCR, the gold standard method, *C. auris* culture, was performed. A total of 56 axilla/groin and 92 environmental specimens were evaluated.

Statistical analysis: Sensitivity, specificity, positive predictive value, negative predictive value, and confidence interval calculation were analyzed using Medcalc stats (https://www.medcalc.org/calc/diagnostic_test.php (accessed on 9 September 2023)).

## 3. Results

### 3.1. Limit of Detection (LoD)

To evaluate the LoD, we tested 20 replicated contrived samples in sterile Amies media, each containing a *C. auris* concentration of 6.77 × 10^−4^ CFU/mL. In all instances, these samples were consistently detected with a mean Ct value of 35.7 ± 1.2 (Table 2). To authentically replicate real testing conditions, we prepared 20 contrived samples using PCR-negative axilla/groin and environmental samples as matrices, respectively. The LoDs were found to be 0.47 CFU/mL for axilla/groin samples and 0.39 CFU/mL for environmental samples (Table 2). Axilla/groin samples displayed a mean Ct value of 35.6 ± 2.0, while environmental samples exhibited a mean Ct value of 33.7 ± 1.9 (Table 2).

### 3.2. Accuracy

The CAURIS-PCR assay demonstrated a remarkable 100% sensitivity (95%CI: 66.37–100%) by accurately detecting all 9 *C. auris* culture-positive samples. In addition, *C. auris* DNA was identified in 9 out of 139 *C. auris* culture-negative samples, resulting in a 50% positive predictive value (95%CI: 34.71–65.29%) and a perfect 100% negative predictive value (95%CI: 97.20–100%). The assay displayed a specificity of 93.53% (95%CI: 88.06–97%). It is worth noting that all culture-negative CAURIS-PCR-positive samples had a Ct value greater than 35 (Table 3).

Specifically, *C. auris* DNA was detected in 1 out of 49 *C. auris* culture-negative axilla/groin samples yielding an 87.50% positive predictive value (95%CI: 50.15–97.99%) and a 100% negative predictive value (95%CI: 92.60–100%) with a specificity of 97.96% (95%CI: 89.15–99.95%). In contrast, in environmental samples, *C. auris* DNA was detected in 8 out of 90 *C. auris* culture-negative cases, illustrating a 20% positive predictive value (95%CI: 11.43–32.63%) and a 100% negative predictive value (95%CI: 95.60–100%) with a specificity of 91.11% (95%CI: 83.23–96.08%) (Table 3). In these validation data, we noted that all culture-negative CAURIS-PCR positive samples had Ct values greater than 35. This observation suggests that a Ct value above 35 may function as an indicator solely for the presence of RT-PCR-detected *C. auris* DNA. The absence of viable yeast in these samples, due to the traditional culture method’s failure to cultivate *C. auris*, lends support to this interpretation.

In addition, the CAURIS-PCR assay demonstrated its capability to detect isolates representing four distinct clades, which are listed in Table 1, further affirming its versatility and applicability.

### 3.3. Analytical Specificity

The primers and probes employed in the CAURIS-PCR assay have previously been validated for the same purpose in two previous publications [16,17]. These studies thoroughly assessed their specificity, and no cross-reactions were reported. Additionally, we conducted testing on *C. auris* closely related fungi, all of which yielded negative results, further affirming the specificity of the primers and probes for *C. auris*.

### 3.4. Implementation of CAURIS-PCR for Surveillance

Since its integration into the Panther Fusion system at our institute in December 2022 until 21 September 2023, a total of 4809 CAURIS-PCR tests were conducted. Among high-risk inpatients subjected to testing, 115 out of 3886 axilla/groin samples tested positive, indicating a colonization rate of approximately 3.0%. Meanwhile, among the 923 environmental samples analyzed, 98 were found to be positive, resulting in a positivity rate of 10.6%. Only two axilla/groin samples had invalid results during the first try, with subsequent retesting producing negative results. Most importantly, the TAT for CAURIS-PCR results was 24 h.

Upon the request of the Infection Control and Healthcare Epidemiology (ICHE) Department, a *C. auris* culture was added to environmental samples that remained CAURIS-PCR-positive after two rounds of bleach cleaning and UV light exposure. Similarly, every CAURIS-PCR-positive axilla/groin swab was subjected to culture to attempt *C. auris* isolation and to send isolates to the Texas State Public Health Laboratory for epidemiology purposes. We analyzed a total of 165 CAURIS-PCR-positive samples that were reflexed to a culture based on ICHE department or state public health laboratory requests. In total, 113 out of 165 (68.5%) CAURIS-PCR-positive samples, which were culture-negative, had a mean Ct value of 36.96 ± 3.21, suggesting that the colony count of viable *C. auris* in the test sample was very low. The remaining 52 (31.5%) samples, which were both CAURIS-PCR and culture-positive, exhibited a mean Ct value of 29.46 ± 5.37. The difference in mean Ct values between these two groups was statistically significant (*p* < 0.0001).

## 4. Discussion

The implementation of a molecular test for the detection of *C. auris* in our laboratory stemmed from a critical necessity to have a highly sensitive and specific test with quick TAT. A direct request from our ICHE department emphasized the urgency of incorporating this test into our facility. Previous studies have detailed assays using traditional real-time PCR, as well as automated systems such as BD Max and Roche Cobas for *C. auris* detection [17,18,19]. Traditional real-time PCR, while effective, demands manual pipetting, making it resource-intensive. The BD Max platform, although beneficial, processes a limited number of 24 samples per batch, rendering it less conducive to our high testing load. Acquiring a Roche Cobas instrument was financially prohibitive. At our laboratory, we have two Panther instruments with Fusion capable of running Open Access samples by real-time PCR. Molecular technologists were already familiar with this instrument, requiring minimal training for the addition of the CAURIS-PCR assay. Moreover, the capability to concurrently perform various assays alongside the CAURIS-PCR assay on the Panther Fusion enhances the Hologic Panther Fusion’s suitability for our specific needs.

In addition, our observed high-risk patient colonization rate of 3.0% aligns with rates reported in other similar studies [6,12,13,20]. Overall, we had a good agreement between culture and CAURIS-PCR, with an exception in the case of environmental samples. Despite rigorous bleach cleaning procedures for all tested surfaces, the prevalence of *C. auris* colonization on healthcare surfaces remained notably high, with over 10% of swabs retaining detectable amounts of *C. auris* DNA even after thorough bleach cleaning. CAURIS-PCR cannot distinguish whether this detectable DNA originates from live yeast capable of colonization and causing infection or from inactivated cells. Nonetheless, the persistence of detectable DNA is concerning since bleach typically destroys DNA [21,22,23]. This highlights the daunting challenge posed by *C. auris* in eradicating its presence from healthcare surfaces.

The relatively low positive predictive value of 20% for environmental samples signifies disparities in results between culture and CAURIS-PCR. This lower positive predictive value for environmental samples compared to the clinical samples could be attributed to differences in collection methods. For instance, one swab was used to sample both the axilla and groin areas from the same patient, while different swabs were used for each spot sampled within the same patient room. The discrepancy between culture and CAURIS-PCR can also be attributed to the inherently lower sensitivity of culture compared to PCR methods. In specimens with a low *C. auris* burden, live yeast cells may go undetected on the agar plate used for culture, even when present in the specimen. Additionally, yeast cells might settle to the bottom of the tubes loaded onto the Hologic instrumentation for CAURIS-PCR, where the low-position aspiration setting could capture DNA from yeast cells missed by culture. Moreover, the high prevalence of CAURIS-PCR-positive culture-negative samples suggests significantly enhanced sensitivity by CAURIS-PCR. This heightened sensitivity is also reflected in the higher mean Ct values of culture-negative CAURIS-PCR-positive specimens compared to both culture- and CAURIS-PCR-positive specimens. While UV light or indirect bleach exposure might hamper the ability of *C. auris* to grow in culture, there is limited research available to determine if non-culturable but viable *C. auris* cells are rendered clinically irrelevant [24,25,26]. Additionally, an important limitation of the LDT employed in our study is the inability to conduct antifungal susceptibility testing or whole-genome sequencing for clade determination and epidemiological studies without an isolate.

The implementation of this LDT significantly reduced TAT from a minimum of 72 h with the culture method to just 24 h, a saving of 2 to 4 days. The 24 h TAT included the idle time used to batch specimens over the weekend, a particularly crucial aspect during weekends with limited staffing. Importantly, this change had no major impact on other routine clinical testing conducted on the Panther Fusion system.

**Figure 1:**
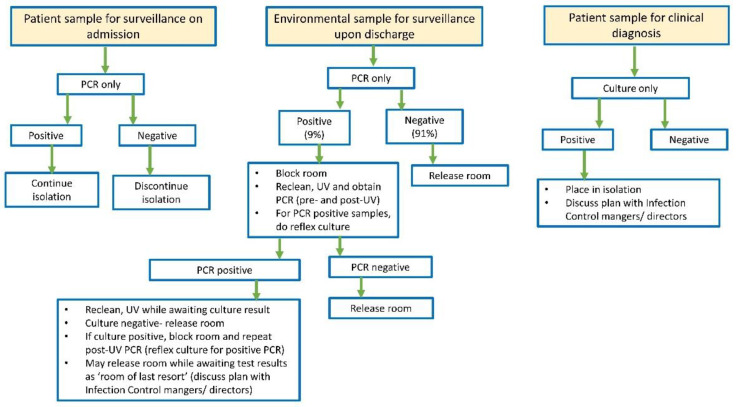
Updated algorithm for *Candida auris* testing, patient isolation, and room decontamination.

Due to the reduction in TAT and enhanced sensitivity of CAURIS-PCR, infection preventionists revised the patient isolation algorithm (Figure 1). For high-risk patients initially screened upon admission, a negative CAURIS-PCR result became a prerequisite before discontinuing isolation precautions. Positive patients continued to be isolated, and stringent room cleaning after discharge remained in effect. The importance of promptly discontinuing unnecessary isolation precautions cannot be overstated, as these patients often receive suboptimal care [27,28,29].

In the context of environmental surveillance for *C. auris*, a rigorous protocol is in place. For a patient room to be cleared for the next occupant, all eight swabs must return negative results. However, if one or more site(s) yield positive results, a meticulous process unfolds. The room undergoes thorough recleaning, followed by UV light treatment and a subsequent round of swabbing. If, upon retesting, any of the eight swabs show positive results for a second time, the ESwab collection is reflexed to culture. In the absence of isolated *C. auris*, the room is released for use. However, if *C. auris* grows in culture, the room undergoes a third round of intensive cleaning and UV light treatment and is designated as a “room of last resort.” This meticulous approach ensures a stringent and proactive response to any potential environmental contamination, emphasizing our commitment to maintaining a safe and infection-free healthcare environment.

While a noticeable gap exists in the literature concerning *C. auris* outbreaks within prison systems, the risks inherent in such environments are conceptually akin to those in LTAC or SNF. These facilities cater to individuals with complex and chronic medical needs and face challenges such as hygiene issues and frequent utilization of broad-spectrum antibiotics. We have now included automated screening for all prisoners being admitted to our prison hospital. Also, given *C. auris*’s ability to persist on surfaces for extended periods, form biofilms, and exhibit resistance to common cleaning solutions used in many hospital systems, these conditions present a fertile ground for potential outbreaks [30,31,32]. Therefore, further research and development for the molecular detection of *C. auris* are needed. Collaborations with industry partners to access commercially available tests could expedite the validation and integration of such tests. In the event of a *C. auris* outbreak, minimizing assay validation and TAT becomes pivotal for effective control of this lethal hospital-associated pathogen, especially in populations that are particularly vulnerable.

## Figures and Tables

**Table 1:** CDC biosample accession number and clade information for each isolate.

Accession Number	Clade
SAMN18754594	African
SAMN05379609	African
SAMN05379619	South American
SAMN05379608	East Asian
SAMN05379624	South Asian

**Table 2:** Limit of detections (LoDs) based on different matrices used for spiking in *C. auris*.

Matrices	LoDs (CFU/mL)	Ct *
Sterile Amies media	6.77 × 10^−4^	35.7 ± 1.2
CAURIS-PCR negative axilla/groin samples	0.47	35.6 ± 2.0
CAURIS-PCR negative environmental samples	0.39	33.7 ± 1.9

* Mean ± standard deviation.

**Table 3:** CAURIS-PCR and *C. auris* culture results in axilla/groin and environmental samples.

			Culture				
			Axilla/Groin		Environment		Total
			Positive	Negative	Positive	Negative	
CAURIS-PCR	Axilla/Groin	Positive	7	1 *	-	-	8
		Negative	0	48	-	-	48
	Environment	Positive	-	-	2	8 *	10
		Negative	-	-	0	82	82
	Total		7	49	2	90	148

* Ct > 35.

## Data Availability

The original contributions presented in the study are included in the article. Further inquiries can be directed to the corresponding authors.
